# When Whole-Genome Alignments Just Won't Work: kSNP v2 Software for Alignment-Free SNP Discovery and Phylogenetics of Hundreds of Microbial Genomes

**DOI:** 10.1371/journal.pone.0081760

**Published:** 2013-12-09

**Authors:** Shea N. Gardner, Barry G. Hall

**Affiliations:** 1 Computations/Global Security, Lawrence Livermore National Laboratory, Livermore, California, United States of America; 2 Bellingham Research Institute, Bellingham, Washington, United States of America; Indiana University, United States of America

## Abstract

Effective use of rapid and inexpensive whole genome sequencing for microbes requires fast, memory efficient bioinformatics tools for sequence comparison. The kSNP v2 software finds single nucleotide polymorphisms (SNPs) in whole genome data. kSNP v2 has numerous improvements over kSNP v1 including SNP gene annotation; better scaling for draft genomes available as assembled contigs or raw, unassembled reads; a tool to identify the optimal value of k; distribution of packages of executables for Linux and Mac OS X for ease of installation and user-friendly use; and a detailed User Guide. SNP discovery is based on k-mer analysis, and requires no multiple sequence alignment or the selection of a single reference genome. Most target sets with hundreds of genomes complete in minutes to hours. SNP phylogenies are built by maximum likelihood, parsimony, and distance, based on all SNPs, only core SNPs, or SNPs present in some intermediate user-specified fraction of targets. The SNP-based trees that result are consistent with known taxonomy. kSNP v2 can handle many gigabases of sequence in a single run, and if one or more annotated genomes are included in the target set, SNPs are annotated with protein coding and other information (UTRs, etc.) from Genbank file(s). We demonstrate application of kSNP v2 on sets of viral and bacterial genomes, and discuss in detail analysis of a set of 68 finished *E. coli* and *Shigella* genomes and a set of the same genomes to which have been added 47 assemblies and four “raw read” genomes of H104:H4 strains from the recent European *E. coli* outbreak that resulted in both bloody diarrhea and hemolytic uremic syndrome (HUS), and caused at least 50 deaths.

## Introduction

SNP phylogenetics play a role in outbreak tracking, forensic investigations, inferring lineage evolution, and identifying mutations linked to phenotypes like drug resistance. Next generation sequencing technologies enable whole genome sequencing of many isolates. kSNP software finds SNPs and builds phylogenies for large numbers of finished and draft sequences. It does not require a reference genome or multiple sequence alignment. Version 2 includes improvements from version 1 [1] for speed, memory efficiency, and SNP annotation.

Usually SNP finding begins with a multiple sequence alignment or many pairwise sequence alignments of a set of target sequences. It is challenging, however, to find software to build accurate alignments for hundreds of microbial genomes in a feasible time frame, particularly if there are multiple distantly-related clades. Mauve [2] is a whole genome multiple alignment program, but its memory demands are such that it cannot align more than 30 bacterial genomes, and aligning 25 genomes requires over 70 hours [3]. Gegens [3] and BopGenomes [4] compare complete genomes without aligning them, but both compare genomes only on the basis of the presence/absence of DNA segments, and do not identify SNPs. Some approaches look for core regions shared among all input genomes, but if the strains are too distantly related (e.g. viral genera) even the core genome may be too divergent to find SNP loci shared by all genomes [5]. Core SNPs exclude loci that are distinguishable within some clades but absent in others. Core SNPs will not cover mutations from regions deleted in a branch of the tree, or SNPs in horizontally transferred genes present in a subset of genomes. Including draft genomes with gaps removes more loci from the core list, which is particularly problematic if there are multiple draft genomes each with gaps in different regions. For successful core SNP analyses, other methods require that genomes are clustered into closely related clades with SNP analyses performed separately for each clade, requiring other analyses to determine the relationships among clades.

Unlike other methods for SNP identification, kSNP v2 can analyze hundreds of bacterial or viral genomes in only a few hours. It can analyze together a set of finished genomes, genome assemblies, and genomes that are at the raw read stage. kSNP is a reference free method, which facilitates analyses of more genomes that are more distantly related than an alignment-based or reference-genome based approach to finding SNPs. kSNP builds Maximum Likelihood, Neighbor Joining, and parsimony phylogenetic trees based on all SNPs, only core SNPs, and SNPs present in at least a user-specified fraction of genomes. Finally, if some finished genomes are included in the data set kSNP annotates the identified SNPs based on the GenBank files of those genomes, which greatly speeds up the process of correlating SNPs with genetic functions. kSNP v2 is available at https://sourceforge.net/projects/ksnp as executables for Mac OS X and for Linux 64-bit operating systems, and as source code; a complete User Guide is provided.

## Methods

### kSNP

**Figure 1:**
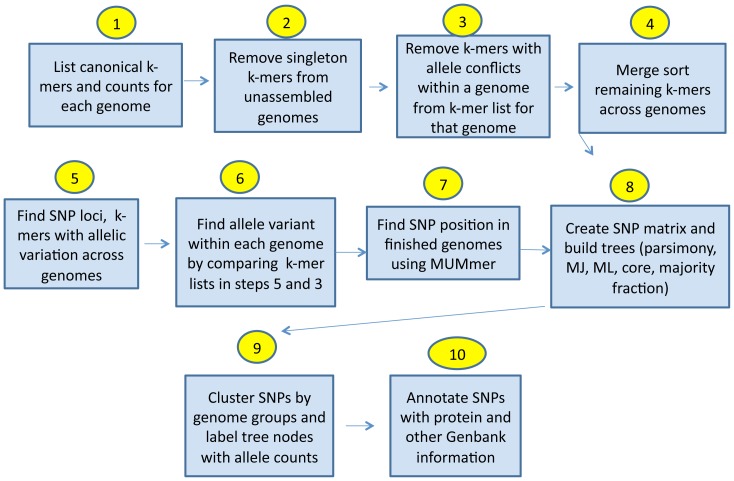
Diagram of the kSNP v2 process.

The user provides a fasta input file of the target genomes for SNP discovery, and specifies k, the length of the flanking sequence including the SNP; i.e. k = 13 means that each SNP will be flanked by 6 bases on each side which are conserved among at least two genomes. The SNP is at the central base of the k-mer, and the flanking (k-1)/2 bases either side of the SNP define the SNP locus. The user also specifies which (if any) genomes are finished, for which SNP positional information is desired. Figure 1 diagrams the procedure. Step 1: The code begins by enumerating all k-mer oligos and their counts separately for each input genome using the open source code jellyfish [6] (http://www.cbcb.umd.edu/software/jellyfish/, version 1.1.2) for k≤31 or sa for k>31 [7], [8]. It condenses the k-mer list and count into canonical k-mers, where only the first in alphabetic sort order of the forward and reverse complement k-mer is retained, with counts reflecting both occurrences on the plus and minus strands. Step 2: for genomes provided as unassembled raw reads, it discards singleton k-mers that occur only once, as these are likely to be sequencing errors. Step 3: For each genome, it then discards k-mers for that genome which would result in a SNP conflict: a SNP conflict results if a single genome has more than one central base variant for a given locus flanking sequence. Step 4: It then does a merge sort across genomes of the conflict-deleted list of k-mers for each genome. Step 5: It looks for SNP loci in the merged list as k-mers in the list for which there are central base variants. Step 6: It compares the SNP loci in step 5 with the conflict-deleted k-mer lists for each genome created in step 3 to determine the allele variants in each genome, and reports the locus (flanking sequence) and allele (central base) for every genome containing that locus. Step 7: SNP positions in the finished genomes are found by matching with MUMmer [9]. This data is output in various formats: SNP allele fasta alignment, SNP matrix, and SNP list with allele positions relative to each genome the user has specified as finished, enabling further analyses by the user.

In step 8, SNP phylogenies are calculated from the SNP allele sequences using several alternative methods: parsimony as implemented by Parsimonator [10], Neighbor Joining of pairwise distances from SNP difference counts [11], and Maximum Likelihood [12]. The reason kSNP_v2 creates so many different kinds of trees (ML, parsimony, Neighbor Joining, core, majority0.5) is that different trees make better sense for different target sets, e.g. viral versus bacterial, how distantly related are the taxa included, whether substitution rates vary among branches, and whether the rate at which sites evolve changes non-identically over time. ML outperforms other methods when substitution rates vary among branches, i.e. ML is less susceptible to long branch attraction bias [13], [14], while maximum parsimony has been shown to outperform ML when the rates at which different sites evolve change at different rates over time [15]. If there are site-specific changes in evolutionary rates over time, known as heterotachy, the non-parametric estimation of trees provided by parsimony is more accurate than a parametric model such as ML. In bacteria with thousands of genes, rapidly evolving genes or SNPs are a small fraction of the genome, so heterotachy probably has a small influence on estimating the tree, and we usually prefer ML SNP trees, which usually result in fewer homoplastic SNPs. That is, we often find that more SNPs map to nodes of the ML tree than the parsimony tree for bacteria. In some viruses, in contrast, evolutionary rates may change more rapidly in genes like envelope and capsid which play a larger role in transmission and immune evasion than do structural or polymerase genes. With small genomes the portions affected by heterotachy can be a large fraction. This could explain why we often find that a parsimony SNP tree seems to most accurately represent that from a full genome multiple sequence alignment and have fewer homoplasies for RNA viruses.

Trees are also calculated with Maximum Likelihood based on all SNPs, core SNPs only for which the loci are present in all the input genomes, and SNPs for which the loci are present in at least a user specified fraction of genomes. In step 9, SNP loci are mapped back onto each tree, and trees are rooted by taking as root the node resulting in the most SNPs mapping to nodes of the tree. Newick formatted tree files are created for importing to a graphics display tool such as Dendroscope [16] or FigTree [17] with branch-specific allele counts plotted at each node and optionally at each leaf. Homoplastic SNPs that do not map exactly to a node of the tree are clustered by the groups of genomes which share alleles. A cluster file reports the genome clusters for each locus and whether the pattern of shared alleles maps to an internal node, a leaf node, and/or a homoplastic group.

In step 10, SNP loci are annotated by cross referencing the SNP positions in finished genomes with the annotations provided by Genbank files which are automatically downloaded from NCBI [18] by gi number. Gene and protein coding information, amino acid sequence, UTRs, and regulatory regions spanning SNP loci are reported. Whether the SNP is synonymous or nonsynonymous is determined, and various summary files are created, such as number of SNPs and NS/S ratio per protein. A script is also included to create a variant call format (VCF) file relative to a reference sequence, optionally run if the –v option is specified. SNPs not present on the reference are not show in the VCF, so users may wish to create VCF files for several reference genomes.

#### Tree formats

Each of the phylogenetic trees is given in four versions, each of which displays different information. (1) The basic tree, e.g. tree.ML.tre, includes no node labels. (2) The AlleleCounts tree, e.g. tree_AlleleCounts.ML.tre, labels the internal nodes with the number of SNPs that are shared exclusively by the descendants of that node. (3) The tipAlleleCounts tree, e.g. tree_tipAlleleCounts.ML.tre, in addition to labeling the internal nodes with the number of SNPs that are shared exclusively by the descendants of that node, also labels the strain names at the tips with the number of SNPs that are exclusive to that strain. (4) The AlleleCounts-NodeLabel tree, e.g. tree_AlleleCounts.ML.NodeLabel.tre, labels the internal nodes with the node number separated by an underscore from the number of SNPs that are shared exclusively by the descendants of that node.

#### Advantages and disadvantages of kSNP

kSNP cannot find SNPs that are too close together (closer than one half k). K is usually in the range of 13–31. For viruses, we have found that k = 13 or 15 works well, and for bacteria, k = 19 or 21, and have included the Kchooser script to assist the user in selecting an optimal value of k for a given data set. Repetitive elements like gene duplications can contain SNPs so long as the duplicate kmer locus does not create an allele conflict *within* a given genome. Even if such regions create allele conflicts within a subset of genomes, the SNP locus can still be detected as a SNP in other genomes without an allele conflict. This facilitates identification of SNPs on regions that may be duplicated or horizontally transferred, such as phage, plasmids, or other mobile elements, in those genomes for which the duplication does not create a SNP allele conflict. But the SNP will not be reported in the genomes with allele conflicts, which would require a longer value of k, i.e. more sequence context, in order to tell the duplicates apart. So running k with a longer value of k should be better at distinguishing loci in homologous regions by detecting some of the SNPs that would be considered allele conflicts with a shorter value of k. But the tradeoff is that a longer k will miss all those high density SNPs in which there is sequence variation within half k of the SNP.

kSNP cannot distinguish true SNPs from sequencing errors. It is advised that for raw read data, some quality filters are imposed on the reads prior to running kSNP (e.g. replace bases with quality below Q20–Q30 with N, and remove adaptors, barcodes, or other non-biological portions of reads). kSNP v2 does not find indels. Indel sequencing errors that occur in the kmer sequence flanking a SNP will cause a SNP detection failure for that locus in that genome.

Some unique features of kSNP v2 are that it scales better for large data sets (hundreds of bacterial or viral genomes) than other SNP finding approaches (Table 1). It can handle many genomes as unassembled raw reads. For example, we have run it in 6.9 hours on 5.8 GB of input for 212 Salmonella genomes, including many in raw reads from multiple sequencing technologies, on a node with 48 GB of RAM and 12 CPU. It does not require a multiple sequence alignment or a reference sequence, so avoids biases stemming from the choice of a reference. kSNP finds SNPs that are not in the core genome, as well as those that are. It phylogenetically analyzes both core SNPs only, and all SNPs, and allows users to investigate cases intermediate between these ends of the spectrum, as SNP loci shared by at least a user-specified fraction of the genomes. One application of kSNP could be a quick initial look at a large data set to determine clades, prior to full genome multiple sequence alignments of genomes within clades to look at strain differences including indels in more detail.

**Table 1:** Optimum values of k for the examples in Table 2.

Target Set	Optimum K	Fraction core kmers at optimum K
Example 1^1^	13	0.067
Example 2^2^	21	0.391
Filoviridae family	15	0.072
Rabies Lyssavirus	13	0.077
Rhabdoviridae family	13	0.018
*Acinetobacter*	19	0.012
*Escherichia coli O104:H4* clade	19	0.36
*Escherichia coli-Shigella* 68 finished genomes	19	0.28
*Escherichia coli-Shigella including O104:H4 strains from European outbreak*	19	0.29

^1^ Example 1 data set (provided with kSNP) consists of 11 equine encephalitis virus finished genomes.

^2^ Example 2 data set provided with kSNP consists of 7 finished, 5 assembled and 2 raw read *E. coli* genomes.

Parallel processes are used at many steps of the code, so multiple CPU are advantageous. The code is for linux/unix operating systems. It is written primarily in PERL and tcsh unix shell script, and requires several other software packages which do not need to be installed separately for the compiled versions of kSNP: MUMmer [9], jellyfish [6], FastTree [12], Parsimonator [10], E-utilities EFetch from NCBI[18], and some bioperl modules. It is open source and freely available from sourceforge.org (https://sourceforge.net/projects/ksnp/files/).

#### Improvements from version 1

For better speed, v2 uses MUMmer instead of BLAST, jellyfish instead of sa (suffix array) for k-mers<32, and FastTreeMP and Parsimonator instead of RAxML [19] and PHYLIP [11]. There are algorithmic changes as well: In version 1, k-mers were initially computed for all genomes at once, and these k-mer lists were used to find candidate SNPs. BLAST was run to compare all candidate k-mers against all genomes to identify SNPs (allele variation among genomes), conflicting alleles (allele variation within a genome), and identify the allele variant within each genome. This use of BLAST was more memory intensive because all candidate SNP loci and all possible allele variants had to be compared to each genome, and positions even in raw read or merged contig genomes were found, even though that positional information was irrelevant. When run against GB of genomes in raw reads in v1, this step was more likely to run out of memory. In version 2, k-mer comparisons are used much more extensively and BLAST is replaced by MUMmer, which is called very minimally. First, jellyfish is run against each genome individually, and PERL and Unix scripts are used to parse the k-mer lists to determine SNPs, alleles within each genome, and conflicting alleles. Forward and reverse complement k-mers and counts are summed and only the orientation occurring first in an alphabetic sort is stored, saving time and space compared to v1. However, this means that more of the loci are reported in the reverse direction than in kSNP v1. MUMmer is only used to determine the position of the allele in finished genomes specified in the -p option input file. Also, k-mer calculations are performed in subsets by prefix, enabling better memory management for extremely large data, and better parallelization.

In addition, SNPs are annotated with information from Genbank files, automatically retrieved from NCBI using the efetch utilities, regarding whether SNPs land on proteins, genes, regulatory regions, etc., and amino acids coded by SNP variants, and various summary files are created. Also, a script is included to create VCF files for a specified reference genome, for compatibility with other tools.

To better handle genomes available only as unassembled raw reads with sequencing errors, the user specifies which genomes are not assembled. k-mers present only once (one may change this threshold in the run script) in the raw reads for a given genome can be removed prior to SNP finding, to avoid considering probable sequencing errors as SNPs or conflicting alleles.

New files beginning with “ClusterInfo” are created for each phylogenetic tree showing, for each SNP, whether it is a core SNP and the node or cluster of genomes sharing alleles, according to the genome-cluster information in the files “Homoplasy_groups” and “Node_SNP_counts”. The ClusterInfo files are handy for parsing, for example, if you want to pull out the loci that map to a given node or homoplastic group or leaf SNPs. A script “select_node_annotations” is provided to pull out the annotated loci mapping to a particular node of a tree.

Estimation of NJ trees is now optional. We noticed that when analyzing a large data set (207 bacterial genomes, 2,376,218 SNPs) it took 26 hours to build the distance matrix from which the NJ tree was computed. This was just over half of the total time for the analysis. Estimation of NJ trees is now optional, invoked by the command line argument –j.

### Simulations

EvolveAGene3 [20] was used to generate simulated data sets for exploring kSNP. The root sequence to initialize EvolveAGene3 simulations was the Eastern equine encephalitis virus strain Georgia 97 (gi|51103286) genome, a sequence of 11,682 base pairs. A random topology that was used for the first simulation was used for all subsequent simulations. In order to vary the amount of sequence diversity the mean branch lengths were varied.

### Minimum spanning trees

Minimum spanning trees were estimated from all of the SNPs by MSTgold based on the equidistant method [21]. MSTgold is freely available at http://bellinghamresearchinstitute.com/software/.

### Phylogenetic tree comparisons

Phylogenetic trees were compared by the program CompareTrees4, a later version of the program described in [22] and freely available at http://bellinghamresearchinstitute.com/software/index.html.

### Congruency

Congruency between phylogenetic trees and their corresponding minimum spanning trees was determined by a perl script, Congruency.pl. Congruency is available as an executable for Mac OS X or Linux by request to BGH at barryghall@gmail.com.

### Unassembled genomes

The E. coli O104:H4 genomes in unassembled raw reads were downloaded from the NCBI Sequence Read Archive (SRA, http://www.ncbi.nlm.nih.gov/Traces/sra) and preprocessed with fastq-dump —split-spot —skip-technical –dumpbase (from NCBI SRA Toolkit, http://www.ncbi.nlm.nih.gov/Traces/sra/sra.cgi?view=software), seqtk trimfq, and

seqtk seq -A -L 25 -l 0 -q 20 -n N (https://github.com/lh3/seqtk) before running the merge_fasta_reads script provided with kSNP. This pre-processing masked bases with quality below Q20 and eliminated reads with length shorter than 25 nt.

## Results and Discussion

### Simulation studies to evaluate the efficiency with which kSNP identifies SNPs

Efficient identification of SNPs is dependent on the correct choice of k, the length of the flanking sequence and SNP used to characterize SNP loci. If k is too small there will non-homologous sequences that match the query kmer by chance alone, resulting in the identification of false SNPs. If k is too large then SNPs will be missed when two SNPs are closer together than the length of the sequence flanking the SNP site in the kmer. The correct choice of k depends on the data set: the longer the genome sequences the larger k must be in order to avoid false SNPs resulting from chance matches. The more sequence variation that is present the smaller k must be in order to avoid missing a significant proportion of the real SNPs.

Because the vast majority of real genomes cannot be aligned, we have used the sequence evolution simulation program EvolveAGene3 to generate data sets for which the true alignment is known, and from which the true number of SNPs can be determined.

We have available just one real data set, a set of 11 Equine Encephalitis virus genomes, for which a robust alignment can be determined. In order to compare a real data set with comparable simulated data sets we used one of those sequence as the root sequence to initiate each simulation. All of the simulations use the same tree topology, and we altered the amount of sequence variation by specifying different mean branch lengths in the simulations. Branch length is the number of changes along that branch divided by the number of sites (bases in the true alignment). Mean branch length is the mean of all the branch lengths.

For each simulated data set we determined the number of true SNPs from the True Alignment, and we used kSNP to estimate the number of SNPs. We define the kSNP efficiency as the number of SNPs identified by kSNP divided by the number of true SNPs.

**Figure 2:**
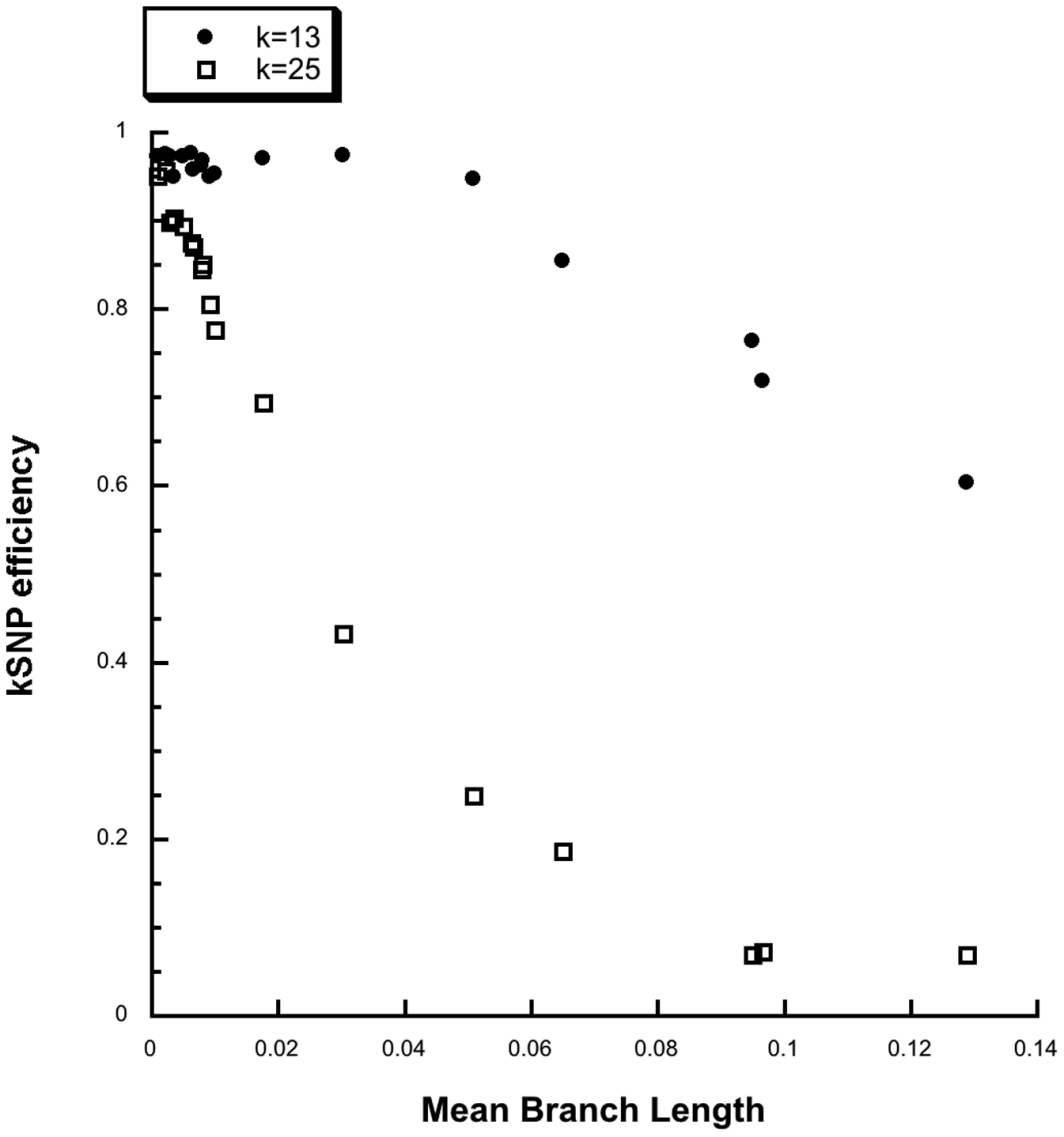
kSNP efficiency vs mean branch lengths of true trees from simulated data sets.

Figure 2 shows that the kSNP efficiency decreases as the mean branch length increases, but that at k = 13 the efficiency remains at around 0.96 until the mean branch length exceeds 0.04, while at k = 25 the efficiency declines very rapidly as branch length increases. This is consistent with our suggestion that as sequence variation increases and the proximity of adjacent SNPs decreases, SNP detection becomes less efficient; and that the larger is k the more serious is the problem.

#### Kchooser, a program to select an optimal k

With real data sets we cannot use alignments to determine the amount of sequence variation, but we reasoned that the greater the amount of sequence variation the smaller would be the number of kmers that are present in every genome (core kmers) at any particular value of k. We also reasoned that as k gets smaller the proportion of core kmers would increase. As k decreases, at some point the number of occurrences of each kmer within one genome will increase as the result of chance matches. Assuming that a genome is a random sequence of length L, the number of times any particular kmer is expected to occur in the same genome by chance alone is L/4^K^, but genomes are anything but random. Some kmers will not be unique within a genome because of duplications and the possible presence of multiple copies of mobile elements within the genome. We are interested in identifying a threshold value of k for which non-unique kmers are the result of real duplication, not the result of chance. The program Kchooser chooses the median length genome from a data set, then tries as the initial value of k an odd integer that would make the chance of a kmer occurring twice <0.01 if the genome were a random sequence. It then counts all of the kmers and the number of times each occurs. On the reasonable assumption that less than 1% of a genome is likely to be duplicated, if the fraction of unique kmers in that genome is <0.99 it increments k by 2 and tries again. If the fraction of unique kmers is >0.99, to err on the side of caution, the optimum value of k is chosen as k+2. Kchooser reports the fraction of unique kmers at each value of k that is tested.

At that optimum value of k, **Kchooser** picks the shortest genome in the data set, identifies all of the kmers in that genome, and determines for each of a random 1000 of those kmers whether it is a core kmer; i.e. is present in all genomes. The shortest genome is selected as the exemplar because all other genomes will include more kmers that are absent in some genomes because the target genome is smaller. **Kchooser** reports the fraction of kmers that are core kmers at the optimum k.

The assumption that less than 1% of a genome is actually duplicated holds well for most data sets that we have considered (see Tables 1 and 2), including the set of 68 *E. coli-Shigella* finished genomes. When that assumption is invalid **Kchooser** continues incrementing k until the maximum value of k = 31 is reached (31 is the largest value used by jellyfish, the program that **Kchooser** uses to enumerate kmers). When that happens we have observed that the fraction of unique kmers reaches a plateau at some lower value of k. So far the lowest fraction of unique kmers that we have observed has been 0.96. We therefore provide an option to specify a lower fraction of unique kmers on the command line in order to permit evaluating the fraction of core kmers in the data set at an appropriate value of k.

**Table 2:** kSNP v2 timings for some examples.

Target Set	Number of sequences	Target set size (MB)	Number of SNP loci^1^	Time to complete (hrs) Linux Cluster^2^	Time to complete (hrs) iMac Desktop^3^
Example 1^4^	11	0.181	943	0.04	0.035
Example 2^5^	14	1,800	63,096	0.43	0.86
Filoviridae family	54	1.1	5,427	0.24	0.33
Rabies Lyssavirus	186	2.2	32,879	0.99	0.98
Rhabdoviridae family	288	3.5	106,381	2.20	2.48
*Acinetobacter*	207	775	2,376,218	35.2	NA
*Escherichia coli O104:H4* clade	57	6,875	35,272	2.11	4.52
*Escherichia coli-Shigella* 68 finished genomes	68	339	418,500	10.6	10.3
*Escherichia coli-Shigella including O104:H4 strains from European outbreak*	119	7,188	430,159	14.4	20.3

^1^ kSNP was run at the optimum setting of k as determined by **Kchooser**. See Table 1.

^2^ Linux cluster: Linux OS TOSS 2.0, 2.8 GHz Xeon EP X5660 processor, 12 cores, 48 GB RAM.

^3^ iMac Desktop: OS X 7.5.3, 3.4 GHz Intel Core i7 processor, 4 cores, 16 GB RAM.

^4^ Example 1 data set (provided with kSNP) consists of 11 equine encephalitis virus finished genomes.

^5^ Example 2 data set provided with kSNP consists of 7 finished, 5 assembled and 2 raw read *E. coli* genomes.

**Figure 3:**
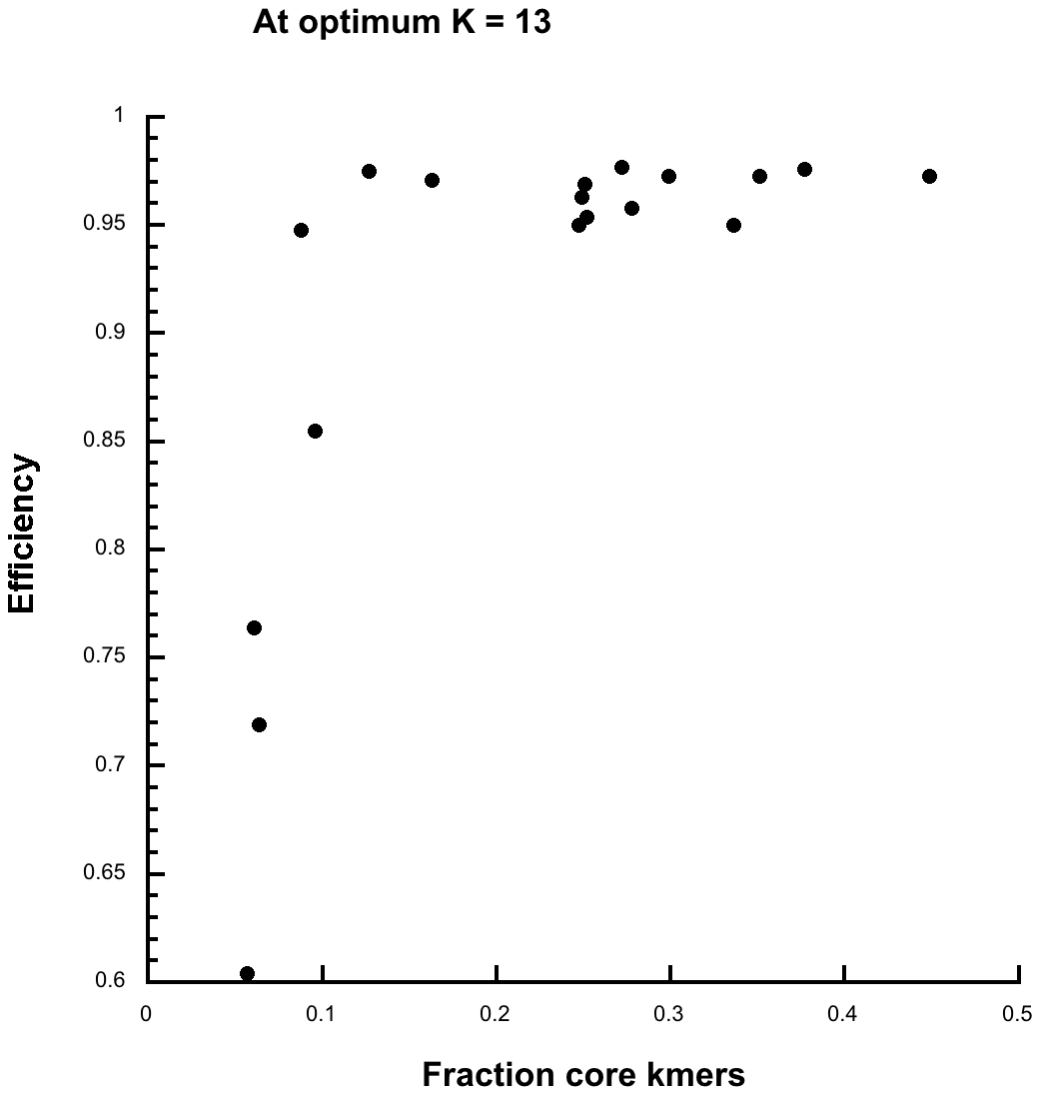
kSNP efficiency vs the fraction of core kmers in simulated data sets.

**Kchooser** was used to determine the optimum value of k and the fraction of core kmers for each of the simulated data sets in Figure 2. Figure 3 shows that as the fraction of core kmers increases kSNP efficiency increases until it reaches a plateau above 10% core kmers. Based on these simulations it seems likely that when the fraction of core kmers is ≥0.1 over 90% of the SNPs will be identified by kSNP. At the optimum k = 13, the fraction of core kmers decreases very regularly as branch length (sequence variation) increases: Fraction of core kmers  = −0.14029−0.19177log(Branch length), R = 0.99246. The fraction of core kmers is thus a good proxy for sequence variation.

#### Consequences of choosing a larger than optimal value of k

Table 1 shows that for all of the viral genomes, and for the *Acinetobacter* genomes, at the optimum value of k the fraction of core kmers is well below 0.1, suggesting that a substantial fraction of the SNPs have not been detected. However, Kchooser indicates that using a lower value of k is likely to result in identifying false SNPs.

When the fraction of core kmers is below 0.1, there is a risk of missing a significant fraction of the SNPs. The importance of that risk depends upon the intended use of the SNPs that are identified. If the purpose is SNP association studies then it must be recognized that some SNPs that are associated with a phenotype may well have been missed. If the purpose is to estimate a phylogenetic tree, it appears to be the case that a sample of SNPs will serve as well as all (or most) of the SNPs. We compared the topologies of the ML trees estimated by kSNP at k = 13 and at k = 25 with the topology of the true tree. In every case, even when kSNP efficiency was only 0.068, the topology of the ML tree was identical with the topology of the true tree. We conclude that phylogenetic tree estimation is very robust with respect to kSNP efficiency and that there is therefore little risk associated with using the optimum k value for tree estimation purposes even when the fraction of core SNPs suggests that the efficiency of SNP finding is likely to be low.

### Time and computational demands of kSNP v2

We have evaluated the time and computational demands of kSNP v2 on two different platforms (Table 2): a 12-core Linux cluster with 48 GB of RAM available, and a 4-core Max OS X platform with 16 GB of RAM available. the data sets ranged from 0.18 MB to 7.2 GB, from 11 viral genomes to 207 bacterial genomes, and the number of SNPs identified ranged from 943 to 2,376,218. The execution times were not significantly different on the two platforms. The Mac OS X desktop completed the SNP discovery and tree building steps for all the target sets, although it exceeded RAM during the step mapping SNPs to nodes of the phylogeny. The approximate RAM requirements can be estimated from the product of the mean genome size (MGS) (excluding genomes consisting of raw reads) and the number of genomes (Figure S1), as Required RAM ∼ = (0.04 *MGS *Num Genomes)−1.5.

### Analysis of 68 Escherichia coli – Shigella genomes

**Figure 4:**
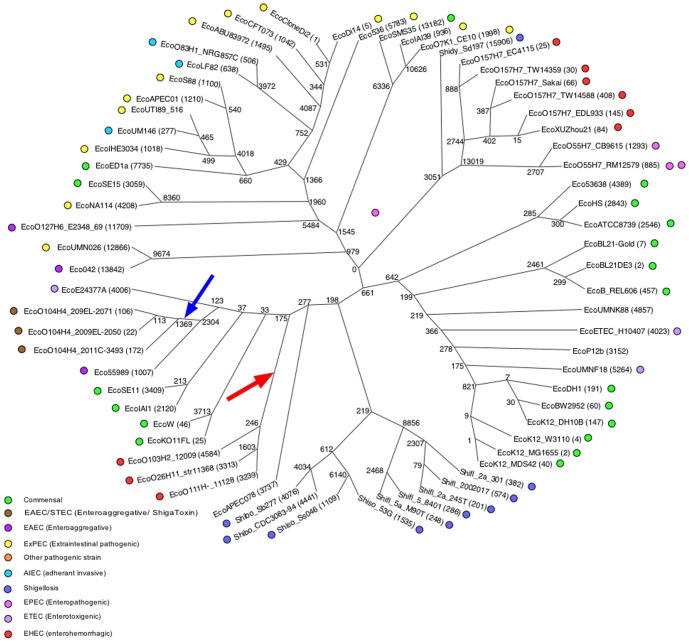
Maximum Likelihood tree of 68 finished *E. coli* genomes. The tree is shown in the radial (unrooted) cladogram format in which branches are drawn without reference to branch lengths. Colored dots indicate pathogenicity phenotype. Arrows indicate branches that can be removed to create particular clusters (see text). Numbers at the internal nodes indicate the number of alleles that are shared exclusively by the descendants of each node. Numbers in parentheses following the genome names are exclusive to that genome.

k was set to 19. Figure 4 shows a Maximum Likelihood tree of 68 *Escherichia coli* and *Shigella* finished genomes in the unrooted radial phylogram format with numbers at the nodes indicating the number of alleles that are shared exclusively by the descendants of each node or that are exclusive to each genome. We point out that all of the trees estimated by kSNP v2 are unrooted trees. GenBank accession numbers and literature citations for phenotypes are in Supporting Information Table S1. Colored dots indicating pathogenicity phenotypes show that in general those phenotypes cluster well. The tree is in good agreement with other trees of the *E. coli-Shigella* group [23].

Phylogenetic analysis is the most widely used tool to estimate biological relationships, but it is often not the most appropriate tool to estimate relationships among bacterial genomes because, as the result of frequent genetic exchange, different parts of the genome can have different evolutionary histories. Phylogenetic analysis assumes that characters that are shared by a pair of individuals are identical by descent. Deviations from that assumption, called *homoplasies*, may arise from convergence, genetic recombination, etc. Homoplasies contribute to a loss of phylogenetic signal and can lead to errors in the topology of a phylogenetic tree. kSNP records the number of homoplastic SNPs on each of the phylogenetic trees. On the ML tree 37.6% of the SNPs are homoplastic.

#### Minimum Spanning Tree (MST)

**Figure 5:**
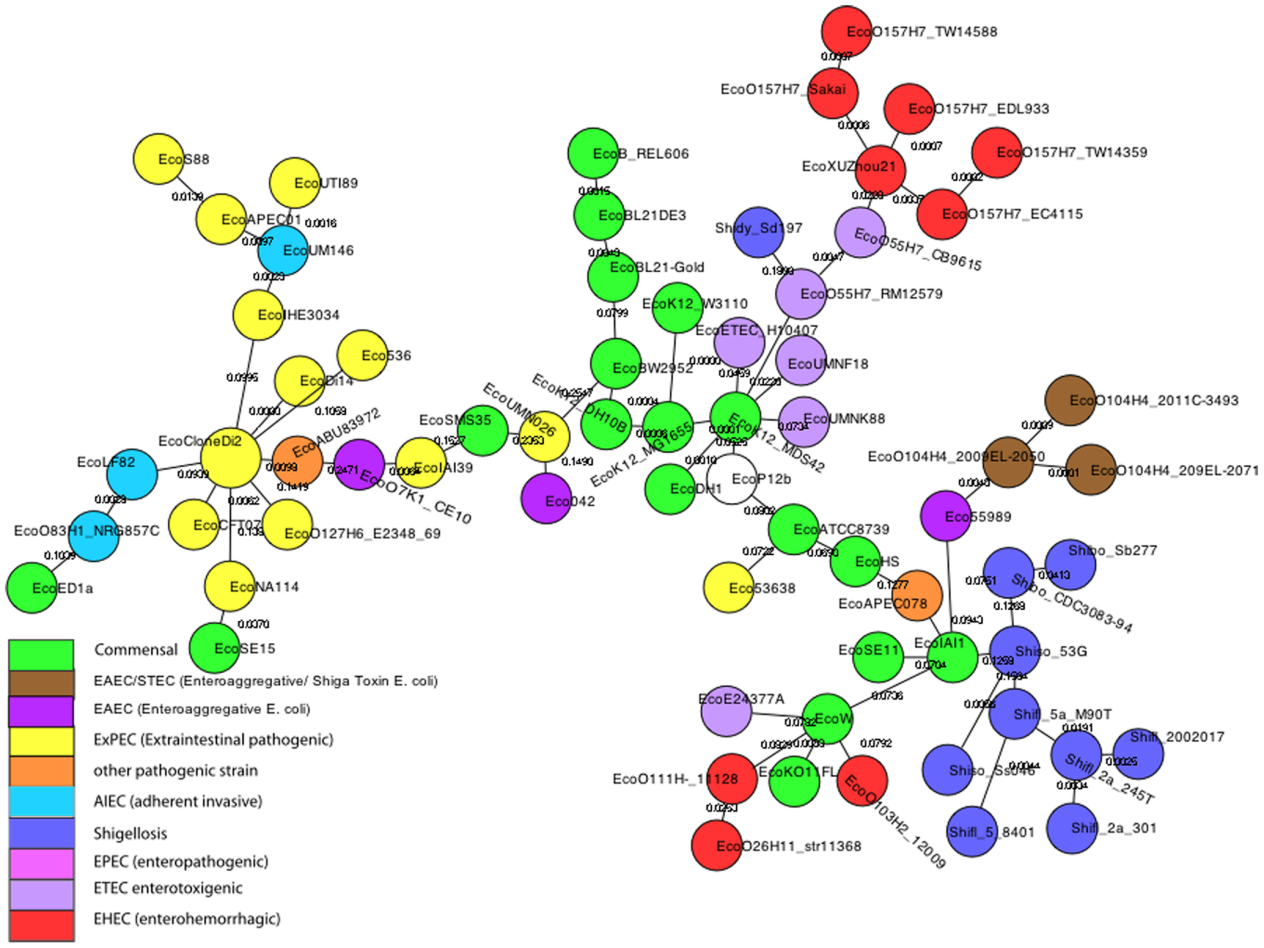
Minimum Spanning Tree of 68 finished *E. coli* genomes. Nodes are colored according to pathogenicity phenotype.

An alternative analysis, by Minimum Spanning Trees (MST), only assumes identity by state; i.e. relationships among genomes are based only upon their similarity, not upon relationships to hypothetical ancestors. An MST is therefore not a different kind of phylogenetic tree, it is an entirely different way of representing relationships among genomes based only on their similarities. Figure 5 shows an MST of the *E. coli- Shigella* genomes based on all of the SNPs. There is generally good agreement between the MST based on SNPs and an MST of most of the same strains based on the presence/absence of DNA fragments [4].

On a phylogenetic tree a cluster is defined as a bipartition that arises by removing a branch. Each of the resulting groups is a cluster. On an MST a cluster is defined as a subgroup such that there is a path from any member of the cluster to any other member without passing through a node that is not a member of the cluster. For instance, removing the branch indicated by the blue arrow on Figure 4 defines a cluster that includes all three O104:H4 genomes. Those genomes also constitute a cluster on the MST (Figure 5), therefore the ML tree cluster is congruent with the MST cluster. In contrast, removing the branch indicated by the red arrow on Figure 4 defines a cluster consisting of EcoO103H2_12009, EcoO111H-_str11128, and EcoO26H11_str11368. That cluster is not congruent with the MST because on the MST the path from EcoO103H2_12009 to EcoO111H-_str11128 passes through EcoW, which is not a member of the cluster on the ML tree.

We can quantify the agreement between a phylogenetic tree and its corresponding MST as a “congruency score” that is the fraction of the clusters on the phylogenetic tree that are also clusters on the MST. The congruency score for the phylogenetic tree in Figure 4 and the MST in Figure 5 is 0.554. This is not to say that either estimate of genome relatedness is better than the other; they estimate different things. Phylogenetic trees estimate relationships to hypothetical ancestors, while MSTs estimate relationships based strictly on similarity of state. If the complete absence of homoplasies phylogenetic trees and MSTs should be 100% congruent.

### Analysis of the *E. coli O104:H4* Europe 2011 outbreak strains

**Figure 6:**
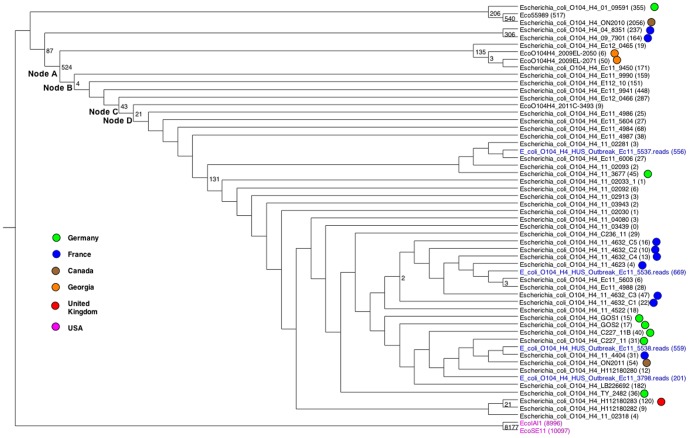
Maximum Likelihood tree of O104:H4 *E. coli* strains. Tree is shown in the rectangular cladogram format and has been rooted with the outgroup consisting of two commensal strains (labeled in magenta). Genomes consisting of raw reads are labeled in blue. Colored dots indicate country of origin where known. Numbers at the internal nodes indicate the number of alleles that are shared exclusively by the descendants of each node. Zeros are not shown. Numbers in parentheses following the genome names are number of alleles exclusive to that genome.

**Figure 7:**
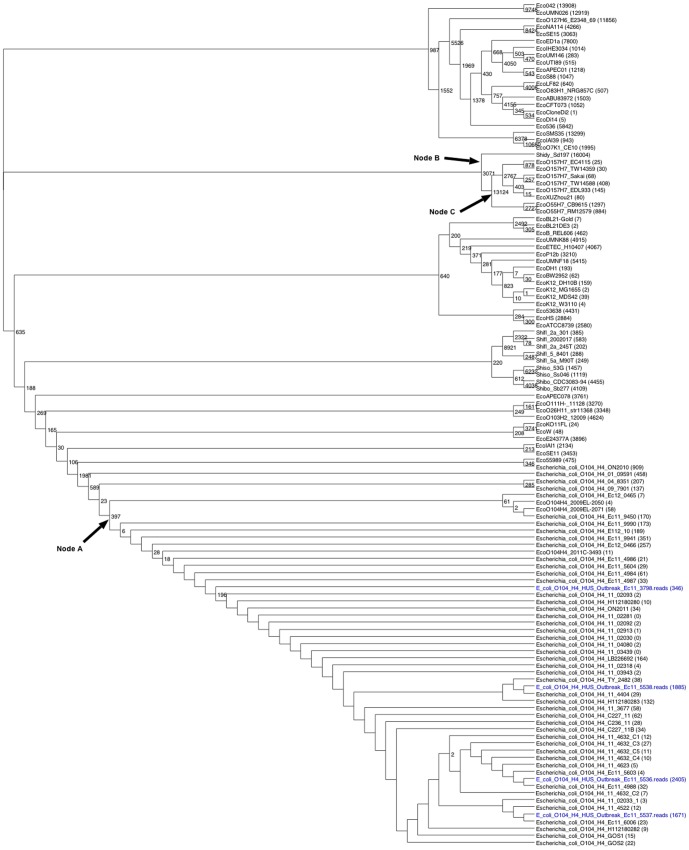
Maximum Likelihood tree of 119 *E. coli* strains. Tree is shown in the rectangular cladogram format, but readers are reminded that this is an unrooted tree. Genomes consisting of raw reads are labeled in blue. Numbers at the internal nodes indicate the number of alleles that are shared exclusively by the descendants of each node. Zeros are not shown. Numbers in parentheses following the genome names are exclusive to that genome. Node A, leading to the 2011-12 European outbreak strains,and nodes B and C, also leading to particularly pathogenic strains, are discussed in the text.

In the summer of 2011 Europe suffered an outbreak of enteroaggregative (EAEC) shiga-toxin producing (STEC) O104:H4 *E. coli* that resulted in 50 deaths, over 800 cases of hemolytic uremic syndrome, and around 4000 cases of bloody diarrhea [24]–[26]. Several studies have examined the genetic relationships among some of those strains and relationships of those strains to other pathogenic *E. coli* strains [23], [25], [26]. At this time, in addition to the three finished O104:H4 genomes, there are 47 assembled genomes and four genomes at the raw read stage available. We take advantage of that availability to assess the relationships among those 54 O104:H4 strains and to assess their relationship to the other 65 *E. coli* and *Shigella* finished genomes by SNP analysis.

k was set to 19. Figure 6 shows a Maximum Likelihood tree of the O104:H4 strains, with the country of origin, where reported, indicated by colored dots. On that tree 5.6% of the SNPs were homoplastic. The tree includes three strains that are not O104:H4: Eco55989 is EAEC, but not STEC and is known to be closely related to the O104:H4 strains ([23] and see Figure 7), strains EcoIAI1 and EcoSE11 are commensal strains and were used as an outgroup to root the tree.

The O104:H4 strains from 2001 and the Ontario Canada 2010 strains cluster with Eco55989. Then the 2004 and 2009_7901 strains branch off together, but do share 87 SNP alleles with the branch leading to the 2011-12 European outbreak strains. The 2009EL strains from Georgia as well as EC12_0465 and EC11_9450 branch off next (Node A), and uniquely share 524 SNPs with the other European outbreak strains. Below that branch on the tree, the other European outbreak E. coli's share a smaller number of SNPs at some nodes, but nowhere near the main divergence point with 524 SNPs.

We compared the SNPs found in [26] with those found by kSNP. Since the fully assembled chromosome was not available at NCBI for the TY2482 genome at the time of our analysis, we only had the draft assembled contigs for this genome in our original analysis of the O104:H4 clade. In order to find the SNP positions corresponding to those reported in [26] we included the 4^th^ assembly of the TY2482 genome from BGI (ftp.genomics.org.cn). We used the O104:H4 strains, but omitted the 3 outgroup strains Eco55989, EcoSE11, and EcoIAI1, and we included both the draft assembly for TY2482 from NCBI and the completed assembly from BGI. All the SNPs from Grad et al. [26] except the SNP at position 2,029,740 were found by kSNP. This SNP was not found because the genomes that have the variant allele when considering a larger context of 80 nt around the SNP have an allele conflict when only the 19-mer context is considered, as the 19-mer occurs twice with a different central base in the genomes that have a different allele than the TY2482 genome. Running kSNP with a longer k might pick up this SNP, although it would also miss others with variable bases in the (k-1)/2 bases around the SNP. Missing this one SNP, however, is far offset by the vastly larger number of SNPs detected by kSNP than the 21 SNPs found in the analysis of just 16 genomes by Grad et al: kSNP detected 4797 SNP loci in the TY-2482 genome, and a total of 8568 SNP loci, 2679 of them core SNPs. The tip SNP counts shown in Figure 6 in parentheses after the strain names indicate that kSNP identified a larger number of isolate-specific alleles than reported in [26]. This could be attributed to a number of factors, including the larger number of genomes in the analysis, reference-free SNP detection so that SNPs relative to genomes other than the TY2482 assembly can be found, and detection of SNPs from unaligned regions of those genomes that would be missed by alignment approaches. These SNPs should be verified that they are in truly homologous regions by aligning a larger sequence context around the SNP.

On Nodes A, B, C, and D in Figure 6, with 524, 4, 43, and 21 SNPs, respectively, for the branches leading to all or most of the 2011–2012 European outbreak strains, the node numbers were determined by looking at the tree plotted from the file tree_AlleleCounts.ML.NodeLabel.tre, and those loci were selected using the select_node_annotations script. These annotations are shown in Table S2. There are 177, 1, 8, and 14 nonsynonymous SNPs for nodes A, B, C, and D, respectively. These include a number of phage related proteins, endolysin, penicillin-binding protein,multidrug efflux system proteins, endolysin, biofilm synthesis proteins, and other genes possibly related to virulence or resistance.

The genomes available as unassembled raw reads had 201-669 genome-specific allele calls, many more than the finished O104:H4 genomes (9-50 genome-specific allele calls) but overlapping the range of the assembled draft genomes from 2011-12 (0-448) genome-specific allele calls). The 2010 strain from Ontario, Canada was very divergent with 2056 strain-specific alleles, and the 2001, 2004, and 2009 O104:H4 strains had 355, 237, and 164, respectively. kSNP cannot distinguish sequencing errors from true SNPs, and errors likely comprise a fraction of the SNPs in draft and unassembled sequences, and potentially a small fraction of SNPs in finished genomes. The SNPs that map to leaves are more likely to be errors than those SNPs shared among multiple strains, either those that map to nodes or those which are homoplastic.

### Analysis of 119 E. coli genomes

k was set to 19. Figure 7 shows the ML tree of the 119 *E. coli* strains that include the strains from Figures 4 and 6. On that tree 37.4% of the SNPs were homoplastic. The O104:H4 genomes from the 2001-12 outbreak in the lower part of the tree all share 397 alleles (Node A, Figure 7). The annotations for those alleles are shown in Table S3, and the 214 nonsynonymous mutations land on many proteins including phage related genes, microcins, a CRISPR-associated protein, and Type VI secretory pathway genes. Genomes down Node B leading to Shidy_Sd197 (Shigella dysenteriae), E. coli O157:H7, and the enteropathogenic O55:H7 genomes uniquely share 3,071 SNP alleles (509 nonsynonymous SNPs), and at Node C the E. coli O157:H7 and O55:H7 uniquely share 13,124 SNP alleles (3,258 nonsynonymous SNPs).

## Conclusions

kSNP performs rapid SNP discovery, phylogeny, and annotation of finished, draft, and unassembled genomes. It can take as input hundreds of microbial genomes, and return results in minutes to hours on a desktop or small cluster. This contrasts with a multiple sequence alignment approach to SNP finding that could require substantially more time and RAM for sequence alignment, or identification of a reference genome which must contain the SNPs reported. Automated SNP annotation as to genes and amino acids make it straightforward to select SNPs on particular genes, identify genes which are SNP hotspots, and list the genes containing SNPs in a particular cluster of genomes.

Sequencing errors cannot be distinguished from true SNPs with kSNP, so predicted SNPs must be verified with other methods, particularly the allele calls unique to one genome. By using all SNPs to build a phylogeny, the effect of sequencing errors should be minimized since the majority of SNPs are expected to be correctly sequenced. Sequencing errors would have a larger effect on trees created from a small subset of SNPs, like those present on only a few genes or a few canonical SNPs, so in that regard kSNP is advantageous.

The optimal length of k differs depending on the level of divergence of the target set. If k is short, within-genome allele conflicts eliminate SNP candidates for repeated, nonhomologous instances of a k-mer with different central bases, or nonhomologous regions may be mis-identified as SNPs simply because they share a short k-mer. If k is long, SNPs in proximity may not be detected. Users are urged to use **Kchooser** to determine the appropriate length of k for their data sets.

Choosing among the trees generated by kSNP also depends on the divergence of the target set. Divergent viruses often work best with NJ or parsimony, while ML trees are usually better for bacteria. We generally select the tree that results in the fewest homoplastic SNPs. The core tree usually gives poor resolution of closely related strains, particularly if the analysis includes many draft genomes. Using the majority tree (-m option) is sometimes a good compromise to allow decent resolution of closely related strains while ignoring the SNP loci present in only a small fraction of genomes, which stand a greater likelihood of being a result of sequencing errors.

kSNP serves a role for fast initial analysis of many genomes to rapidly create a SNP phylogeny from whole genomes. It could be followed for higher accuracy SNP finding by sequence alignment of small clades whose relatedness is determined from kSNP, or by selecting a small number of genomes for analysis chosen to represent the major phylogenetic branches identified by kSNP.

kSNP is available at https://sourceforge.net/projects/ksnp.

## Supporting Information

Figure S1 **Maximum RAM required by kSNP versus input data size, as required by the runs reported in this paper on the linux cluster.**
(PDF)

Table S1 GenBank accession numbers and literature citations for phenotypes of finished E.coli and Shigella genomes in Figure 2.
(DOCX)

Table S2 SNP annotations mapping to the nodes indicated in Figure 6.
(XLSX)

Table S3 SNP annotations mapping to the node with 397 SNP alleles uniquely shared by the European 2011–2012 outbreak in Figure 7.
(XLSX)
