# Correction: When Whole-Genome Alignments Just Won’t Work: kSNP v2 Software for Alignment-Free SNP Discovery and Phylogenetics of Hundreds of Microbial Genomes

**DOI:** 10.1371/journal.pone.0118258

**Published:** 2015-03-03

**Authors:** The PLOS ONE Staff

As the result of a bug in Kchooser, values of Fraction of Core kmers (FCK) were under-calculated by about a factor of two. As a result, the following sections of the paper were affected. The authors have uploaded a bug-fix version of Kchooser to SourceForge (https://sourceforge.net/projects/ksnp), along with an explanation of the issue.

In the Results and Discussion section, under the “Kchooser, a program to select an optimal k” subsection, there is an error in the third to final sentence and the second to final sentence. In the third to final sentence, “≥0.1” should read “>0.2.” In the second to final sentence, “-0.14029–0.19177log(Branch length), R = 0.99246” should read “-0.24507–0.34278 log(Branch length), R = 0.99265.” The correct sentences are as follow: “Based on these simulations it seems likely that when the fraction of core kmers is >0.2 over 90% of the SNPs will be identified by kSNP. At the optimum k = 13, the fraction of core kmers decreases very regularly as branch length (sequence variation) increases: Fraction of core kmers = -0.24507–0.34278 log(Branch length), R = 0.99265.”

Also in the Results and Discussion section, under the “Consequences of choosing a larger than optimal value of k” subsection, there is an error in the first and third sentences. The number 0.1 should read 0.2. The correct first sentence is as follows: “Table 1 shows that for all of the viral genomes, and for the *Acinetobacter* genomes, at the optimum value of k the fraction of core kmers is well below 0.2, suggesting that a substantial fraction of the SNPs have not been detected.” The correct third sentence is as follows: “When the fraction of core kmers is below 0.2, there is a risk of missing a significant fraction of the SNPs.”

Additionally, there are errors in the “Fraction core kmers at optimum K” column of Table 1. Please view the correct Table 1 below.

**Table 1:** Optimum values of k for the examples in Table 2.

Target Set	Optimum K	Fraction core kmersat optimum K
Example 1^1^	13	0.124
Example 2^2^	21	0.666
Filoviridae family	15	0.128
Rabies Lyssavirus	13	0.146
Rhabdoviridae family	13	0.030
*Acinetobacter*	19	0.021
*Escherichia coli O104*:*H4* clade	19	0.793
*Escherichia coli-Shigella* 68 finished genomes	19	0.525
*Escherichia coli-Shigella including O104*:*H4 strains from European outbreak*	19	0.522

^1^Example 1 data set (provided with kSNP) consists of 11 equine encephalitis virus finished genomes.

^2^Example 2 data set provided with kSNP consists of 7 finished, 5 assembled and 2 raw read *E*. *coli* genomes.

Further, Fig. 3 is incorrect. Please view the correct Fig. 3 here.

**Fig. 3:**
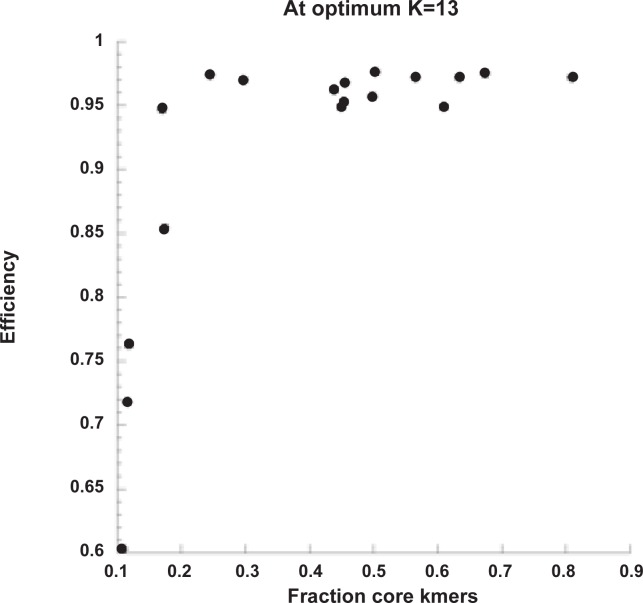
kSNP efficiency vs the fraction of core kmers in simulated data sets.
