# Supplementary figures and images for: When Whole-Genome Alignments Just Won't Work: kSNP v2 Software for Alignment-Free SNP Discovery and Phylogenetics of Hundreds of Microbial Genomes
Source: PLoS One. 2013 Dec 9;8(12):e81760. doi: 10.1371/journal.pone.0081760 (PMC3857212; doi:10.1371/journal.pone.0081760)

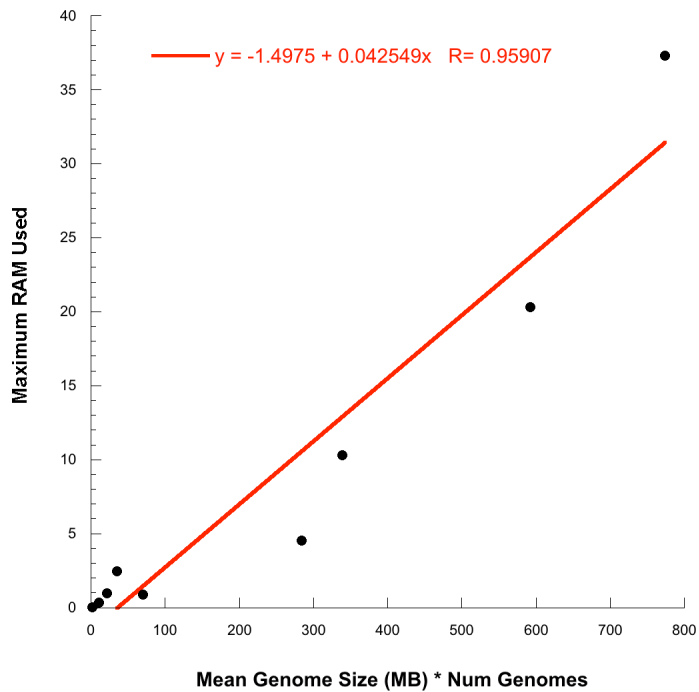

Supplement: Figure S1 — Maximum RAM required by kSNP versus input data size, as required by the runs reported in this paper on the linux cluster. (PDF) [file pone.0081760.s001.pdf]
