# Supplementary material for: When Whole-Genome Alignments Just Won't Work: kSNP v2 Software for Alignment-Free SNP Discovery and Phylogenetics of Hundreds of Microbial Genomes
Source: PLoS One. 2013 Dec 9;8(12):e81760. doi: 10.1371/journal.pone.0081760 (PMC3857212; doi:10.1371/journal.pone.0081760)
Supplement: Table S1 — GenBank accession numbers and literature citations for phenotypes of finished E.coli and Shigella genomes in Figure 2. (DOCX) [file pone.0081760.s002.docx]

**Table S1 Strains, accession numbers and phenotypes**

| **Strain** | **ID on trees** | **Accession Number** | **Phenotype**  **and reference^a^** |
| --- | --- | --- | --- |
| **Finished Genomes** | | | |
| [Escherichia coli 042](http://www.ncbi.nlm.nih.gov/bioproject/40647) | Eco042 | [FN554766](http://www.ncbi.nlm.nih.gov/nuccore/FN554766) | EAEC ([Crossman, et al. 2010](#_ENREF_6)) |
| [Escherichia coli 536](http://www.ncbi.nlm.nih.gov/bioproject/16235) | Eco536 | NC_008253.1 | ExPec UTI ([Zhou, et al. 2010](#_ENREF_38)) |
| Escherichia coli 53638 | Eco53638 | AAKB00000000 | EIEC JCVI (http://gsc.jcvi.org/  projects/msc/e_coli_and_shigella  /escherichia_coli_53638  /index.shtml) |
| [Escherichia coli 55989](http://www.ncbi.nlm.nih.gov/bioproject/33413) | Eco55989 | [NC_011748.1](http://www.ncbi.nlm.nih.gov/nuccore/NC_011748.1) | EAEC ([Sims and Kim 2011b](#_ENREF_27)) |
| [Escherichia coli ABU 83972](http://www.ncbi.nlm.nih.gov/bioproject/38725) | EcoABU83972 | [CP001671](http://www.ncbi.nlm.nih.gov/nuccore/CP001671) | ExPec UTI ([Zdziarski, et al. 2010](#_ENREF_37)) |
| [Escherichia coli APEC O1](http://www.ncbi.nlm.nih.gov/bioproject/16718) | EcoAPEC01 | [NC_008563.1](http://www.ncbi.nlm.nih.gov/nuccore/NC_008563.1) | ExPec From bird ([Johnson, et al. 2007](#_ENREF_12)) ([Zhou, et al. 2010](#_ENREF_38)) |
| Escherichia coli APEC 0[78](http://www.ncbi.nlm.nih.gov/bioproject/16718) | EcoAPEC078 | NC_020163.1 | APEC ([Mangiamele, et al. 2013](#_ENREF_17))) |
| [Escherichia coli ATCC 8739](http://www.ncbi.nlm.nih.gov/bioproject/18083) | EcoATCC8739 | [NC_010468.1](http://www.ncbi.nlm.nih.gov/nuccore/NC_010468.1) | Commensal ([Archer, et al. 2011](#_ENREF_3)) |
| [Escherichia coli B str. REL606](http://www.ncbi.nlm.nih.gov/bioproject/18281) | EcoB_REL606 | [NC_012967.1](http://www.ncbi.nlm.nih.gov/nuccore/NC_012967.1) | Commensal ([Sims and Kim 2011a](#_ENREF_26)) |
| [Escherichia coli 'BL21-Gold(DE3)pLysS AG'](http://www.ncbi.nlm.nih.gov/bioproject/30681) | EcoBL21-Gold | [NC_012947.1](http://www.ncbi.nlm.nih.gov/nuccore/NC_012947.1) | Commensal |
| [Escherichia coli BL21(DE3)](http://www.ncbi.nlm.nih.gov/bioproject/28965) | EcoBL21DE3 | [AM946981](http://www.ncbi.nlm.nih.gov/nuccore/AM946981) | Commensal ([Sims and Kim 2011a](#_ENREF_26)) |
| [Escherichia coli BW2952](http://www.ncbi.nlm.nih.gov/bioproject/33775) | EcoBW2952 | [NC_012759.1](http://www.ncbi.nlm.nih.gov/nuccore/NC_012759.1) | Commensal ([Ferenci, et al. 2009](#_ENREF_7)) |
| Escherichia coli CFT073 | EcoCFT073 | [NC_004431.1](http://www.ncbi.nlm.nih.gov/nuccore/NC_004431.1) | ExPec ([Zhou, et al. 2010](#_ENREF_38)) |
| Escherichia coli str. 'clone D i2' | EcoCloneDi2 | CP002212 | ExPec UPEC ([Reeves, et al. 2011](#_ENREF_24)) |
| Escherichia coli str. 'clone D i14' | EcoCloneDi14 | CP002212 | ExPec UPEC ([Reeves, et al. 2011](#_ENREF_24)) |
| Escherichia coli DH1 | EcoDH1 | [CP001637](http://www.ncbi.nlm.nih.gov/nuccore/CP001637) | Commensal ([Suzuki, et al. 2011](#_ENREF_28)) |
| Escherichia coli E24377A | EcoE24377A | [NC_009801.1](http://www.ncbi.nlm.nih.gov/nuccore/NC_009801.1) | ETEC |
| Escherichia coli ED1a | EcoED1a | NC_011745.1 | Commensal ([Zhou, et al. 2010](#_ENREF_38)) |
| Escherichia coli ETEC H10407 | EcoETEC_H10407 | FN649414 | ETEC ([Zhou, et al. 2010](#_ENREF_38)) |
| Escherichia coli HS | EcoHS | NC_009800.1 | Commensal ([Zhou, et al. 2010](#_ENREF_38)) |
| Escherichia coli IAI1 | EcoIAI1 | NC_011741.1 | Commensal ([Zhou, et al. 2010](#_ENREF_38)) |
| Escherichia coli IAI39 | EcoIAI39 | NC_011750.1 | ExPec UPEC ([Touchon, et al. 2009](#_ENREF_30); [Zhou, et al. 2010](#_ENREF_38)) |
| Escherichia coli IHE3034 | EcoIHE3034 | CP001969 | ExPec Neonatal meningitis ([Moriel, et al. 2010](#_ENREF_19)) |
| Escherichia coli str. K-12 substr. DH10B | EcoK12_DH10B | NC_010473.1 | Commensal ([Zhou, et al. 2010](#_ENREF_38)) |
| Escherichia coli str. K-12 substr. MDS42 | EcoK12_MDS42 | NC_020518.1 | Commensal |
| Escherichia coli str. K-12 substr. MG1655 | EcoK12_MG1655 | NC_000913.2 | Commensal ([Sims and Kim 2011b](#_ENREF_27)) |
| Escherichia coli str. K-12 substr. W3110 | EcoK12_W3110 | AP009048 | Commensal ([Sims and Kim 2011a](#_ENREF_26)) |
| Escherichia coli KO11FL | EcoKO11FL | CP002516 | Commensal ([Turner, et al. 2012](#_ENREF_31)) |
| Escherichia coli LF82 | EcoLF82 | CU651637 | AIEC ([Wine, et al. 2009](#_ENREF_33)) |
| Escherichia coli NA114 | EcoNA114 | CP002797 | ExPec UPEC ([Avasthi, et al. 2011](#_ENREF_4)) |
| Escherichia coli O103:H2 str. 12009 | EcoO103H2_12009 | NC_013353.1 | EHEC ([Sims and Kim 2011a](#_ENREF_26)) |
| Escherichia coli O104:H4 2009EL-2050 | EcoO104H4_2009EL-2050 | CP003297.1 | EAEC/STEC ([Ahmed, et al. 2012](#_ENREF_1)) |
| Escherichia coli O104:H4 2009EL-2071 | EcoO104H4_2009EL-2071 | CP003301.1 | EAEC/STEC ([Ahmed, et al. 2012](#_ENREF_1)) |
| Escherichia coliO104:H4 2011C-3493 | EcoO104H4_2011C-3493 | CP003289.1 | EAEC/STEC ([Ahmed, et al. 2012](#_ENREF_1)) |
| Escherichia coli O111:H- str. 11128 | EcoO111H-_11128 | NC_013364.1 | EHEC ([Sims and Kim 2011a](#_ENREF_26)) |
| Escherichia coli O127:H6 str. E2348/69 | EcoO127H6_E2348_ | NC_011601.1 | EPEC ([Zhou, et al. 2010](#_ENREF_38)) |
| Escherichia coli O157:H7 str. EC4115 | EcoO157H7_EC4115 | NC_011353.1 | EHEC ([Sims and Kim 2011a](#_ENREF_26)) |
| Escherichia coli O157:H7 str. EDL933 | EcoO157H7_EDL933 | NC_002655.2 | EHEC ([Sims and Kim 2011a](#_ENREF_26)) |
| Escherichia coli O157:H7 str. Sakai | EcoO157H7_Sakai | NC_002695.1 | EHEC ([Sims and Kim 2011a](#_ENREF_26)) |
| Escherichia coli O157:H7 str. TW14359 | EcoO157H7_TW14359 | NC_013008.1 | EHEC ([Sims and Kim 2011a](#_ENREF_26)) |
| Escherichia coli O157H7 str. TW14588 | EcoO157H7_TW14588 | [CM000662.1](http://www.ncbi.nlm.nih.gov/nuccore/CM000662.1) | EHEC ([Kulasekara, et al. 2009](#_ENREF_14)) |
| Escherichia coli O26:H11 str. 11368 | EcoO26H11_str11368 | NC_013361.1 | EHEC ([Sims and Kim 2011a](#_ENREF_26)) |
| Escherichia coli O55:H7 str. CB9615 | EcoO55H7_CB9615 | NC_013941.1 | EPEC ([Zhou, et al. 2010](#_ENREF_38)) |
| Escherichia coli O55:H7 str. RM12579 | EcoO55H7_RM12579 | [CP003109.1](http://www.ncbi.nlm.nih.gov/nuccore/CP003109.1) | EPEC ([Kyle, et al. 2012](#_ENREF_15)) |
| Escherichia coli O7:K1 str. CE10 | EcoO7K1_CE10 | CP003034 | ExPec Neonatal meningitis Lu et al 2011 |
| Escherichia coli O83:H1 str. NRG 857C | EcoO83H1_NRG857C | CP001855 | AIEC ([Allen, et al. 2008](#_ENREF_2)) |
| [Escherichia coli P12b](http://www.ncbi.nlm.nih.gov/genome/167?project_id=162061) | EcoP21B | [CP002291.1](http://www.ncbi.nlm.nih.gov/nuccore/CP002291.1) | ????? ([Liu, et al. 2012](#_ENREF_16)) |
| Escherichia coli S88 | EcoS88 | NC_011742.1 | ExPec Neonatal Meningitis ([Touchon, et al. 2009](#_ENREF_30)) ([Zhou, et al. 2010](#_ENREF_38)) |
| Escherichia coli SE11 | EcoSE11 | NC_011415.1 | Commensal ([Zhou, et al. 2010](#_ENREF_38)) |
| Escherichia coli SE15 | EcoSE15 | AP009378 | Commensal ([Toh, et al. 2010](#_ENREF_29)) |
| Escherichia coli SMS-3-5 | EcoSMS35 | NC_010498.1 | Commensal ([Zhou, et al. 2010](#_ENREF_38)) |
| Escherichia coli UM146 | EcoUM146 | CP002167 | AIEC, Ileal Chron's Disease biopsy tissue ([Krause, et al. 2011](#_ENREF_13)) |
| Escherichia coli UMN026 | EcoUMN026 | NC_011751.1 | ExPec UPEC ([Touchon, et al. 2009](#_ENREF_30)) ([Zhou, et al. 2010](#_ENREF_38)) |
| Escherichia coli UMNF18 | EcoUMNF18 | NZ_AGTD01000001.1 | ETEC (([Shepard, et al. 2012](#_ENREF_25))) |
| Escherichia coli UMNK88 | EcoUMNK88 | CP002729 | ETEC ([Fernandez-Alarcon, et al. 2011](#_ENREF_8); [Shepard, et al. 2012](#_ENREF_25)) |
| Escherichia coli UTI89 | EcoUTI89 | NC_007946.1 | ExPec UPEC ([Zhou, et al. 2010](#_ENREF_38)) |
| Escherichia coli W | EcoW | CP002185 | Commensal |
| Escherichia coli Xuzhou21 | EcoXUZhou21 | \|  \| CP001925.1 \| \| --- \| --- \| | EHEC ([Xiong, et al. 2012](#_ENREF_34)) |
| Shigella boydii CDC 3083-94 | Shibo_CDC3083-94 | NC_010658.1 | ([Pupo, et al. 2000](#_ENREF_22)) |
| Shigella boydii Sb227 | Shibo_Sb277 | NC_007613.1 | ([Yang, et al. 2005](#_ENREF_35)) |
| Shigella dysenteriae Sd197 | Shidy_Sd197 | NC_007606.1 | ([Yang, et al. 2005](#_ENREF_35)) |
| Shigella flexneri 2002017 | Shifl_2002017 | NC_004741.1 | ([Ye, et al. 2010](#_ENREF_36)) |
| Shigella flexneri 2a str. 2457T | Shifl_2a_245T | NC_004741.1 | ([Wei, et al. 2003](#_ENREF_32)) |
| Shigella flexneri 2a str. 301 | Shifl_2a_301 | NC_004337.2 | ([Jin, et al. 2002](#_ENREF_11)) |
| Shigella flexneri 5 str. 8401 | Shifl_5_8401 | NC_008258.1 | ([Nie, et al. 2006](#_ENREF_20)) |
| Shigella flexneri 5a str. M90T | Shifi_M90T | CM001474.1 | ([Onodera, et al. 2012](#_ENREF_21)) |
| Shigella sonnei Ss046 | Shiso_Ss046 | NC_008258.1 | ([Yang, et al. 2005](#_ENREF_35)) |
| Shigella sonnei 53G | Shiso_53G | HE616528.1 |  |

^a^If no reference is given the genome is a direct submission and the phenotype is taken from the GenBank file annotation

| **Genome assemblies**  **All are EAEC/STEC** | | | |
| --- | --- | --- | --- |
| **Strain** | **ID on trees** | **Accession Number** | **Country of Origin**  **and reference^a^** |
| Escherichia coli O104:H4 str. 01-09591 | Escherichia_coli_O104_H4_01_09591 | NZ_AFPS00000000.1 | Germany  ([Mellmann, et al. 2011](#_ENREF_18)) |
| Escherichia coli O104:H4 str. 04-8351 | Escherichia_coli_O104_H4_04_8351 | NZ_AFRL00000000.1 | France  ([Grad, et al. 2012](#_ENREF_9)) |
| Escherichia coli O104:H4 str. 09-7901 | Escherichia_coli_O104_H4_09_7901 | NZ_AFRK00000000.1 | France  ([Grad, et al. 2012](#_ENREF_9)) |
| Escherichia coli O104:H4 str. 11-02030 | Escherichia_coli_O104_H4_11_02030 | NZ_AMVR00000000.1 |  |
| Escherichia coli O104:H4 str. 11-02033-1 | Escherichia_coli_O104_H4_11_02033 | NZ_AMVS00000000.1 |  |
| Escherichia coli O104:H4 str. 11-02092 | Escherichia_coli_O104_H4_11_02092 | NZ_AMVT00000000.1 |  |
| Escherichia coli O104:H4 str. 11-02093 | Escherichia_coli_O104_H4_11_02093 | NZ_AMVU00000000.1 |  |
| Escherichia coli O104:H4 str. 11-02281 | Escherichia_coli_O104_H4_11_02281 | NZ_AMVV00000000.1 |  |
| Escherichia coli O104:H4 str. 11-02318 | Escherichia_coli_O104_H4_11_02318 | NZ_AMVW00000000.1 |  |
| Escherichia coli O104:H4 str. 11-02913 | Escherichia_coli_O104_H4_11_02913 | NZ_AMVX00000000.1 |  |
| Escherichia coli O104:H4 str. 11-03439 | Escherichia_coli_O104_H4_11_03439 | NZ_AMVY00000000.1 |  |
| Escherichia coli O104:H4 str. 11-03943 | Escherichia_coli_O104_H4_11_03943 | NZ_AMWA00000000.1 |  |
| Escherichia coli O104:H4 str. 11-3677 | Escherichia_coli_O104_H4_11_3677 | NZ_AFRM00000000.1 | Germany  ([Grad, et al. 2012](#_ENREF_9)) |
| Escherichia coli O104:H4 str. 11-04080 | Escherichia_coli_O104_H4_11_04080 | NZ_AMVZ00000000.1 |  |
| Escherichia coli O104:H4 str. 11-4404 | Escherichia_coli_O104_H4_11_4404 | NZ_AFUX00000000.1 | France  ([Grad, et al. 2012](#_ENREF_9)) |
| Escherichia coli O104:H4 str. 11-4522 | Escherichia_coli_O104_H4_11_4522 | NZ_AFUY00000000.1 | France  ([Grad, et al. 2012](#_ENREF_9)) |
| Escherichia coli O104:H4 str. 11-4623 | Escherichia_coli_O104_H4_11_4623 | NZ_AFUZ00000000.1 | France  ([Grad, et al. 2012](#_ENREF_9)) |
| Escherichia coli O104:H4 str. 11-4632 C1 | Escherichia_coli_O104_H4_11_4632_C1 | NZ_AFVA00000000.1 | France  ([Grad, et al. 2012](#_ENREF_9)) |
| Escherichia coli O104:H4 str. 11-4632 C2 | Escherichia_coli_O104_H4_11_4632_C2 | NZ_AFVB00000000.1 | France  ([Grad, et al. 2012](#_ENREF_9)) |
| Escherichia coli O104:H4 str. 11-4632 C3 | Escherichia_coli_O104_H4_11_4632_C3 | NZ_AFVC00000000.1 | France  ([Grad, et al. 2012](#_ENREF_9)) |
| Escherichia coli O104:H4 str. 11-4632 C4 | Escherichia_coli_O104_H4_11_4632_C4 | NZ_AFVD00000000.1 | France  ([Grad, et al. 2012](#_ENREF_9)) |
| Escherichia coli O104:H4 str. 11-4632 C5 | Escherichia_coli_O104_H4_11_4632_C5 | NZ_AFVE00000000.1 | France  ([Grad, et al. 2012](#_ENREF_9)) |
| Escherichia coli O104:H4 str. C227-11 | Escherichia_coli_O104_H4_C227_11 | NZ_AFST00000000.1 | Denmark/Germany  ([Rasko, et al. 2011](#_ENREF_23)) |
| Escherichia coli O104:H4 str. C227-11 | Escherichia_coli_O104_H4_C227_11B | NZ_AFRH00000000.1 | Denmark/Germany  ([Grad, et al. 2012](#_ENREF_9)) |
| Escherichia coli O104:H4 str. C236-11 | Escherichia_coli_O104_H4_C236_11 | NZ_AFRI00000000.1 | Germany  ([Grad, et al. 2012](#_ENREF_9)) |
| Escherichia coli O104:H4 str. E112/10 | Escherichia_coli_O104_H4_E112_10 | NZ_AHAV00000000. |  |
| Escherichia coli O104:H4 str. Ec11-4984 | Escherichia_coli_O104_H4_Ec11_4984 | NZ_AHOU00000000.1 |  |
| Escherichia coli O104:H4 str. Ec11-4986 | Escherichia_coli_O104_H4_Ec11_4986 | NZ_AHOW00000000.1 |  |
| Escherichia coli O104:H4 str. Ec11-4987 | Escherichia_coli_O104_H4_Ec11_4987 | NZ_AHOX00000000.1 |  |
| Escherichia coli O104:H4 str. Ec11-4988 | Escherichia_coli_O104_H4_Ec11_4988 | NZ_AHOY00000000.1 |  |
| Escherichia coli O104:H4 str. Ec11-5603 | Escherichia_coli_O104_H4_Ec11_5603 | NZ_AHOZ00000000.1 |  |
| Escherichia coli O104:H4 str. Ec11-5604 | Escherichia_coli_O104_H4_Ec11_5604 | NZ_AHOV00000000.1 |  |
| Escherichia coli O104:H4 str. Ec11-6006 | Escherichia_coli_O104_H4_Ec11_6006 | NZ_AHPA00000000.1 |  |
| Escherichia coli O104:H4 str. Ec11-9450 | Escherichia_coli_O104_H4_Ec11_9450 | NZ_AGWF00000000.1 |  |
| Escherichia coli O104:H4 str. Ec11-9941 | Escherichia_coli_O104_H4_Ec11_9941 | NZ_AGWH00000000.1 |  |
| Escherichia coli O104:H4 str. Ec11-9990 | Escherichia_coli_O104_H4_Ec11_9990 | NZ_AGWG00000000.1 |  |
| Escherichia coli O104:H4 str. Ec12-0465 | Escherichia_coli_O104_H4_Ec12_0465 | NZ_AIPQ00000000.1 |  |
| Escherichia coli O104:H4 str. Ec12-0466 | Escherichia_coli_O104_H4_Ec12_0466 | NZ_AIPR00000000.1 |  |
| Escherichia coli O104:H4 str. GOS1 | Escherichia_coli_O104_H4_GOS1 | NZ_AFWO00000000.1 | Germany  ([Brzuszkiewicz, et al. 2011](#_ENREF_5)) |
| Escherichia coli O104:H4 str. GOS2 | Escherichia_coli_O104_H4_GOS2 | NZ_AFWP00000000.1 | Germany  ([Brzuszkiewicz, et al. 2011](#_ENREF_5)) |
| Escherichia coli O104:H4 str. H112180280 | Escherichia_coli_O104_H4_H112180280 | NZ_AFPN00000000.2 |  |
| Escherichia coli O104:H4 str. H112180282 | Escherichia_coli_O104_H4_H112180282 | NZ_AFSO00000000.1 |  |
| Escherichia coli O104:H4 str. H112180283 | Escherichia_coli_O104_H4_H112180283 | NZ_AFWC00000000.1 | United Kingdom |
| Escherichia coli O104:H4 str. LB226692 | Escherichia_coli_O104_H4_LB226692 | NZ_AFOB00000000.2 | Germany  ([Mellmann, et al. 2011](#_ENREF_18)) |
| Escherichia coli O104:H4 str. ON2010 | Escherichia_coli_O104_H4_ON2010 | NZ_AHZE00000000.1 | Canada  ([Hao, et al. 2012](#_ENREF_10)) |
| Escherichia coli O104:H4 str. ON2011 | Escherichia_coli_O104_H4_ON2011 | NZ_AHZF00000000.1 | Canada  ([Hao, et al. 2012](#_ENREF_10)) |
| Escherichia coli O104:H4 str. TY-2482 | Escherichia_coli_O104_H4_TY_2482 | NZ_AFVR00000000.1 |  |

^a^If no reference is given the genome is a direct submission and the country of origin is not specified or is taken from the GenBank file.

**References**

Reference

Ahmed SA, Awosika J, Baldwin C, et al. 2012. Genomic comparison of Escherichia coli O104:H4 isolates from 2009 and 2011 reveals plasmid, and prophage heterogeneity, including shiga toxin encoding phage stx2. PLoS One 7: e48228

Allen CA, Niesel DW, Torres AG 2008. The effects of low-shear stress on Adherent-invasive Escherichia coli. Environ Microbiol 10: 1512-1525

Archer CT, Kim JF, Jeong H, Park JH, Vickers CE, Lee SY, Nielsen LK 2011. The genome sequence of E. coli W (ATCC 9637): comparative genome analysis and an improved genome-scale reconstruction of E. coli. BMC Genomics 12: 9

Avasthi TS, Kumar N, Baddam R, Hussain A, Nandanwar N, Jadhav S, Ahmed N 2011. Genome of multidrug-resistant uropathogenic Escherichia coli strain NA114 from India. J Bacteriol 193: 4272-4273

Brzuszkiewicz E, Thurmer A, Schuldes J, et al. 2011. Genome sequence analyses of two isolates from the recent Escherichia coli outbreak in Germany reveal the emergence of a new pathotype: Entero-Aggregative-Haemorrhagic Escherichia coli (EAHEC). Arch Microbiol 193: 883-891

Crossman LC, Chaudhuri RR, Beatson SA, et al. 2010. A commensal gone bad: complete genome sequence of the prototypical enterotoxigenic Escherichia coli strain H10407. J Bacteriol 192: 5822-5831

Ferenci T, Zhou Z, Betteridge T, Ren Y, Liu Y, Feng L, Reeves PR, Wang L 2009. Genomic sequencing reveals regulatory mutations and recombinational events in the widely used MC4100 lineage of Escherichia coli K-12. J Bacteriol 191: 4025-4029

Fernandez-Alarcon C, Singer RS, Johnson TJ 2011. Comparative genomics of multidrug resistance-encoding IncA/C plasmids from commensal and pathogenic Escherichia coli from multiple animal sources. PLoS One 6: e23415

Grad YH, Lipsitch M, Feldgarden M, et al. 2012. Genomic epidemiology of the Escherichia coli O104:H4 outbreaks in Europe, 2011. Proc Natl Acad Sci U S A 109: 3065-3070

Hao W, Allen VG, Jamieson FB, Low DE, Alexander DC 2012. Phylogenetic incongruence in E. coli O104: understanding the evolutionary relationships of emerging pathogens in the face of homologous recombination. PLoS One 7: e33971

Jin Q, Yuan Z, Xu J, et al. 2002. Genome sequence of Shigella flexneri 2a: insights into pathogenicity through comparison with genomes of Escherichia coli K12 and O157. Nucleic Acids Res 30: 4432-4441

Johnson TJ, Kariyawasam S, Wannemuehler Y, et al. 2007. The genome sequence of avian pathogenic Escherichia coli strain O1:K1:H7 shares strong similarities with human extraintestinal pathogenic E. coli genomes. J Bacteriol 189: 3228-3236

Krause DO, Little AC, Dowd SE, Bernstein CN 2011. Complete genome sequence of adherent invasive Escherichia coli UM146 isolated from Ileal Crohn's disease biopsy tissue. J Bacteriol 193: 583

Kulasekara BR, Jacobs M, Zhou Y, et al. 2009. Analysis of the genome of the Escherichia coli O157:H7 2006 spinach-associated outbreak isolate indicates candidate genes that may enhance virulence. Infect Immun 77: 3713-3721

Kyle JL, Cummings CA, Parker CT, et al. 2012. Escherichia coli serotype O55:H7 diversity supports parallel acquisition of bacteriophage at Shiga toxin phage insertion sites during evolution of the O157:H7 lineage. J Bacteriol 194: 1885-1896

Liu B, Hu B, Zhou Z, Guo D, Guo X, Ding P, Feng L, Wang L 2012. A novel non-homologous recombination-mediated mechanism for Escherichia coli unilateral flagellar phase variation. Nucleic Acids Res 40: 4530-4538

Mangiamele P, Nicholson B, Wannemuehler Y, Seemann T, Logue CM, Li G, Tivendale KA, Nolan LK 2013. Complete genome sequence of the avian pathogenic Escherichia coli strain APEC O78. Genome Announc 1: e0002613

Mellmann A, Harmsen D, Cummings CA, et al. 2011. Prospective genomic characterization of the German enterohemorrhagic Escherichia coli O104:H4 outbreak by rapid next generation sequencing technology. PLoS One 6: e22751

Moriel DG, Bertoldi I, Spagnuolo A, et al. 2010. Identification of protective and broadly conserved vaccine antigens from the genome of extraintestinal pathogenic Escherichia coli. Proc Natl Acad Sci U S A 107: 9072-9077

Nie H, Yang F, Zhang X, et al. 2006. Complete genome sequence of Shigella flexneri 5b and comparison with Shigella flexneri 2a. BMC Genomics 7: 173

Onodera NT, Ryu J, Durbic T, Nislow C, Archibald JM, Rohde JR 2012. Genome sequence of Shigella flexneri serotype 5a strain M90T Sm. J Bacteriol 194: 3022

Pupo GM, Lan R, Reeves PR 2000. Multiple independent origins of Shigella clones of Escherichia coli and convergent evolution of many of their characteristics. Proc Natl Acad Sci U S A 97: 10567-10572.

Rasko DA, Webster DR, Sahl JW, et al. 2011. Origins of the E. coli strain causing an outbreak of hemolytic-uremic syndrome in Germany. N Engl J Med 365: 709-717

Reeves PR, Liu B, Zhou Z, et al. 2011. Rates of mutation and host transmission for an Escherichia coli clone over 3 years. PLoS One 6: e26907

Shepard SM, Danzeisen JL, Isaacson RE, Seemann T, Achtman M, Johnson TJ 2012. Genome sequences and phylogenetic analysis of K88- and F18-positive porcine enterotoxigenic Escherichia coli. J Bacteriol 194: 395-405

Sims GE, Kim SH 2011a. Whole-genome phylogeny of Escherichia coli/Shigella group by feature frequency profiles (FFPs). Proceedings of the National Academy of Sciences of the United States of America 108: 8329-8334

Sims GE, Kim SH 2011b. Whole-genome phylogeny of Escherichia coli/Shigella group by feature frequency profiles (FFPs). Proc Natl Acad Sci U S A 108: 8329-8334

Suzuki S, Ono N, Furusawa C, Ying BW, Yomo T 2011. Comparison of sequence reads obtained from three next-generation sequencing platforms. PLoS One 6: e19534

Toh H, Oshima K, Toyoda A, et al. 2010. Complete genome sequence of the wild-type commensal Escherichia coli strain SE15, belonging to phylogenetic group B2. J Bacteriol 192: 1165-1166

Touchon M, Hoede C, Tenaillon O, et al. 2009. Organised genome dynamics in the Escherichia coli species results in highly diverse adaptive paths. PLoS Genet 5: e1000344

Turner PC, Yomano LP, Jarboe LR, York SW, Baggett CL, Moritz BE, Zentz EB, Shanmugam KT, Ingram LO 2012. Optical mapping and sequencing of the Escherichia coli KO11 genome reveal extensive chromosomal rearrangements, and multiple tandem copies of the Zymomonas mobilis pdc and adhB genes. J Ind Microbiol Biotechnol 39: 629-639

Wei J, Goldberg MB, Burland V, et al. 2003. Complete genome sequence and comparative genomics of Shigella flexneri serotype 2a strain 2457T. Infect Immun 71: 2775-2786

Wine E, Ossa JC, Gray-Owen SD, Sherman PM 2009. Adherent-invasive Escherichia coli, strain LF82 disrupts apical junctional complexes in polarized epithelia. BMC Microbiol 9: 180

Xiong Y, Wang P, Lan R, et al. 2012. A novel Escherichia coli O157:H7 clone causing a major hemolytic uremic syndrome outbreak in China. PLoS One 7: e36144

Yang F, Yang J, Zhang X, et al. 2005. Genome dynamics and diversity of Shigella species, the etiologic agents of bacillary dysentery. Nucleic Acids Res 33: 6445-6458

Ye C, Lan R, Xia S, et al. 2010. Emergence of a new multidrug-resistant serotype X variant in an epidemic clone of Shigella flexneri. J Clin Microbiol 48: 419-426

Zdziarski J, Brzuszkiewicz E, Wullt B, et al. 2010. Host imprints on bacterial genomes--rapid, divergent evolution in individual patients. PLoS Pathog 6: e1001078

Zhou Z, Li X, Liu B, et al. 2010. Derivation of Escherichia coli O157:H7 from its O55:H7 precursor. PLoS One 5: e8700
